# Correction: Does behavioral thermal tolerance predict distribution pattern and habitat use in two sympatric Neotropical frogs?

**DOI:** 10.1371/journal.pone.0246851

**Published:** 2021-02-04

**Authors:** Juan C. Díaz-Ricaurte, Filipe C. Serrano, Estefany Caroline Guevara-Molina, Cybele Araujo, Marcio Martins

The affiliations for the second and fifth author are incorrect. Filipe C. Serrano and Marcio Martins are not affiliated with #2 but with #3: Departamento de Ecologia, Instituto de Biociências, Universidade de São Paulo, São Paulo, São Paulo, Brazil.
