# Supplementary material for: Does behavioral thermal tolerance predict distribution pattern and habitat use in two sympatric Neotropical frogs?
Source: PLoS One. 2020 Sep 22;15(9):e0239485. doi: 10.1371/journal.pone.0239485 (PMC7508379; doi:10.1371/journal.pone.0239485)
Supplement: S1 Fig — P. nattereri (A–B) and P. cuvieri (C–D), showing the inner and outer metatarsal tubercles in the detail. Note the much larger and strongly keratinized tubercles in P. nattereri. Photos not to scale. (PDF) [file pone.0239485.s001.pdf]

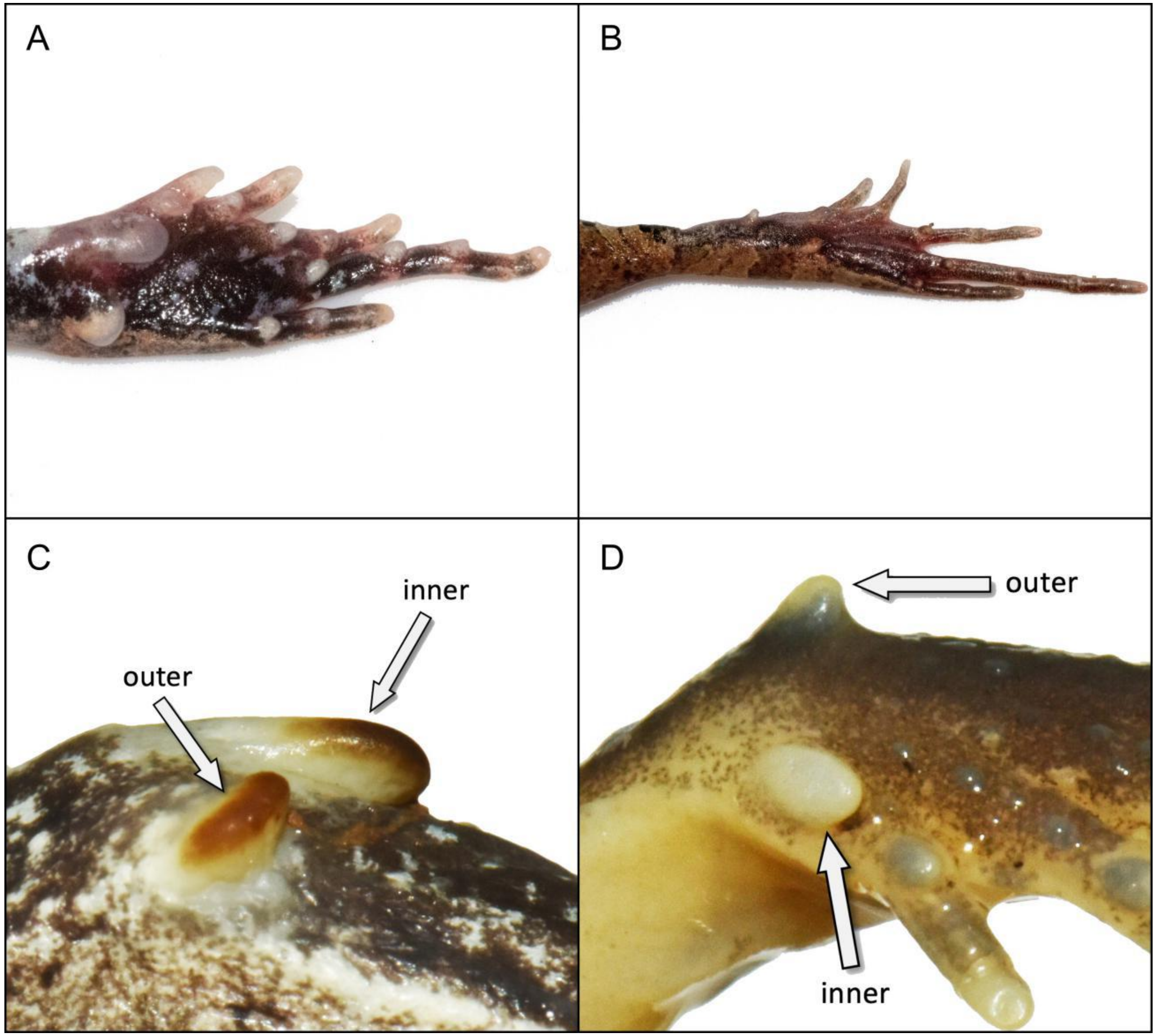

S1 Fig. Detail of hind feet of A-B) *P. nattereri* and C-D) *P. cuvieri*, showing the inner and outer metatarsal tubercles in the detail. Note the much larger and strongly keratinized tubercles in *P. nattereri.* Photos not to scale.
