# Supplementary material for: Does behavioral thermal tolerance predict distribution pattern and habitat use in two sympatric Neotropical frogs?
Source: PLoS One. 2020 Sep 22;15(9):e0239485. doi: 10.1371/journal.pone.0239485 (PMC7508379; doi:10.1371/journal.pone.0239485)
Supplement: S2 Fig — Relative abundance (in %) of P. cuvieri (blue circles) and P. nattereri (red circles) in open (brown) and non-open (green) areas in Cerrado (see S5 Table). The localities are: Floresta Nacional (FLONA) de Silvânia (GO), Reserva Particular do Patrimônio Natural (RPPN) Cabeceira do Prata (MS), Estação Ecológica (EE) Jataí (SP), Estação Ecológica de Itirapina (SP), Estação Ecológica de Santa Bárbara (SP), and Aporé River (GO and MS). Sources of data: [45–50]. Detailed data on the abundance of the frogs in different vegetation types are in S5 Table. (PDF) [file pone.0239485.s002.pdf]

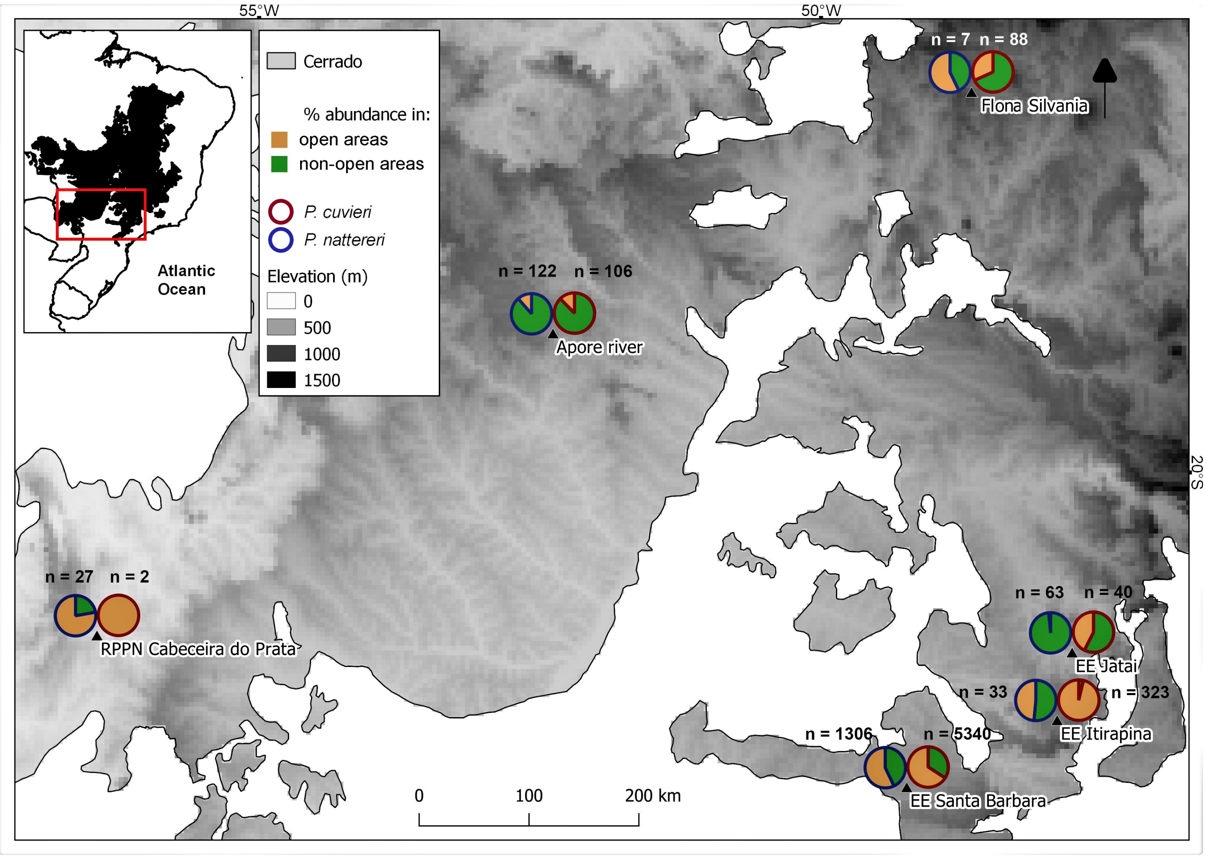

S2 Fig. Relative abundance (in %) of *P. cuvieri* (blue circles) and *P. nattereri* (red circles) **in open** (brown) **and non-open** (green) **areas in Cerrado** (see S5 Table). The localities are: Floresta Nacional (FLONA) de Silvânia (GO), Reserva Particular do Patrimônio Natural (RPPN) Cabeceira do Prata (MS), Estação Ecológica (EE) Jataí (SP), Estação Ecológica de Itirapina (SP), Estação Ecológica de Santa Bárbara (SP), and Aporé River (GO and MS). Sources of data: [45–50]. Detailed data on the abundance of the frogs in different vegetation types are in S5 Table.
