# Supplementary material for: Does behavioral thermal tolerance predict distribution pattern and habitat use in two sympatric Neotropical frogs?
Source: PLoS One. 2020 Sep 22;15(9):e0239485. doi: 10.1371/journal.pone.0239485 (PMC7508379; doi:10.1371/journal.pone.0239485)
Supplement: S3 Fig — A) Temperature measured with sensors buried in the soil at superficial soil (green) and below ground level (red) and in a frog-sized plaster model (blue). B) Illustration of the measurement setup. (PDF) [file pone.0239485.s003.pdf]

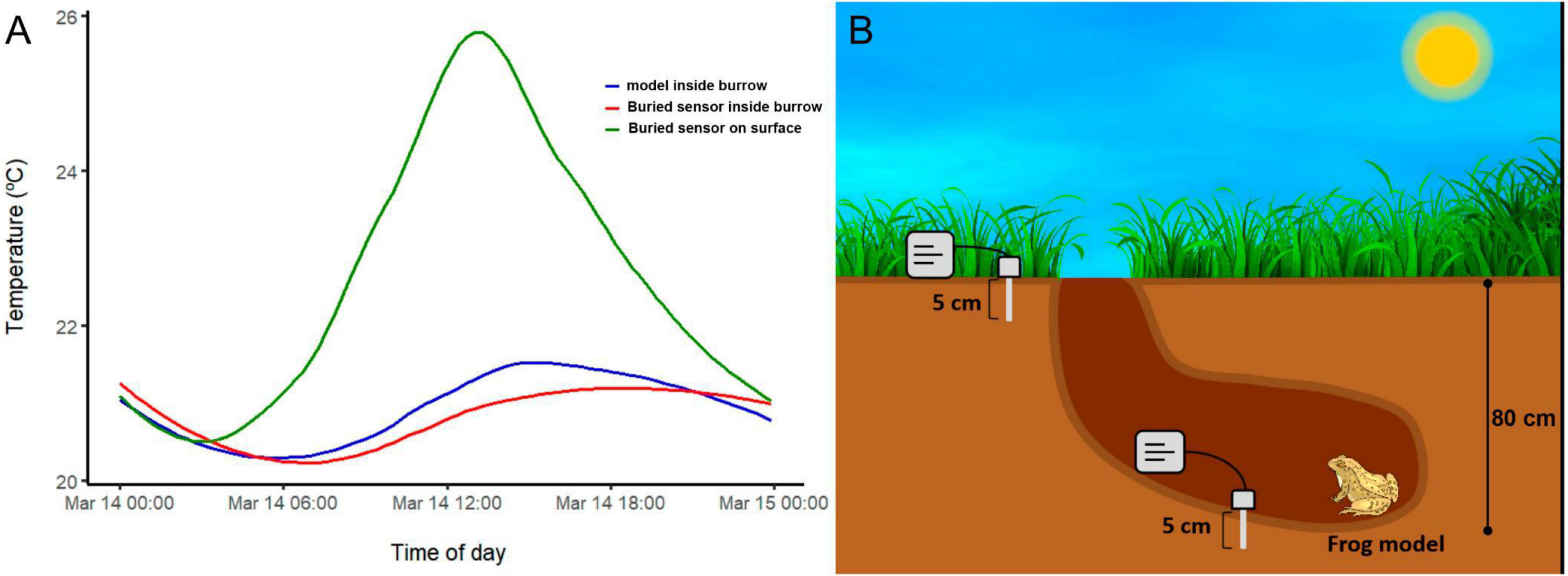

S2 Fig. (A) Temperature during a 24-hour cycle measured with sensors buried in the soil at superficial soil (green) and below ground level (red) and in a frog-sized plaster model (blue). (B) Illustration of the measurement setup.
