# Supplementary material for: Does behavioral thermal tolerance predict distribution pattern and habitat use in two sympatric Neotropical frogs?
Source: PLoS One. 2020 Sep 22;15(9):e0239485. doi: 10.1371/journal.pone.0239485 (PMC7508379; doi:10.1371/journal.pone.0239485)
Supplement: Attachment — Submitted filename: Response letter_RV2_.docx [file pone.0239485.s010.docx]

**Dear Dr. Stefan Lötters**

**Academic Editor**

**PLOS ONE**

We thank the Academic Editor and reviewer #1 for their time and attention in reviewing our manuscript. The comments and suggestions provided by reviewer #1 resulted in changes that enhanced the scientific quality of our manuscript. We have accepted all of the grammatical changes and suggestions. For each comment, we made all the respective corrections, with detailed answers specifying the number of the lines in which the text was changed. If you find that our response is not clear, you can also check the changes in the manuscript version with track changes.

**REVIEWERS' COMMENTS:**

**Reviewer 1:**

**General considerations:**

The authors have satisfactorily addressed the comments from the previous review round. The manuscript is thorough and clear with the exception of very specific parts. Below are my comments on the aspects that must be improved.

**Comment 1:** Maybe the authors could also discuss how sampling bias can affect the results. This is because there is a sampling bias towards the southern portion of the Cerrado biome (see Diniz-Filho et al. 2005; Costa et al. 2007).

**R:** We thank the reviewer for calling our attention for this problem. We added a phrase in the discussion section to clarify that the distribution of our study species is unlikely to be prone to a strong sampling bias due to their easy detectability. See lines 337-341.

**Specific comments:**

**Comment 2-Line 60**: regional and global scales

**R:** Changed as suggested, see line 60.

**Comment 3-Line 65**: the current global warming crisis

**R:** Changed as suggested, see line 65,

**Comment 4-Line 86**: a behavioral thermal tolerance measure

**R:** Changed as suggested, see line 86.

**Comment 5-Line 95**: portray

**R:** Changed as suggested. see line 95,

**Comment 6-Line 105:** for being...

**R:** Changed as suggested, see line 105.

**Comment 7-Lines 107-112:** Why not to mention the species that thte authors know has a larger body size (P. nattereri). I also wonder why not directly referring to "open habitats" as having higher environmental temperature.

**R:** Changed as suggested. see lines 108-110.

**Comment 8-Line 128:** These two species

**R:** Changed as suggested, see line 128,

**Comment 9-Line 142**: The landscape not only consist of...

R: Changed as suggested, see line 142.

**Comment 10-Line 191:** occurence records

**R:** Changed as suggested, see line 191.

**Comment 11-Lines 217:** The model with the highest weight was the null model. I suggest the authors to rephrase this sentence so that it sound more clear and honest.

**R:** Changed as suggested. We have rewritten this sentence to make it clearer that the null model was the chosen one; see lines 216-218.

**Comment 12-Line 235:** was markedly

**R:** Changed as suggested, see line 238.

**Comment 13-Line 241-243:** It is not clear whether P. cuvieri occur in areas where the maximum temperature is lower or higher thans its VTmax

**R:** We agree with the reviewer. We rephrased it to make it clearer, see line 245.

**Comment 14-Lines 250-259:** The results presentend in this part of the text should be summarized through charts. It was difficult to withdraw conclusions from Figure 2. I suggest that Figure 2 should be included in the Supplementary material and a chart (barplots or boxplots) should be presented along the manuscript instead.

**R:** We agree with the reviewer that the figure was not easy to interpret and have changed to barplots, as suggested. Thus, we also have moved the previous Figure 2 (map) to the Supplementary Material (S2 Fig).

**Comment 15-Line 330**: tend to be abundant in both open and non-open areas

**R:** Changed as suggested, see line 336.
